# Supplementary material for: Expression Pattern and Ligand Binding Characteristics Analysis of Chemosensory Protein SnitCSP2 from Sirex nitobei
Source: Insects. 2023 Jun 27;14(7):583. doi: 10.3390/insects14070583 (PMC10380366; doi:10.3390/insects14070583)
Supplement: Supplementary file 1 [file insects-14-00583-s001.zip › Supplementary File/FigS.pdf]

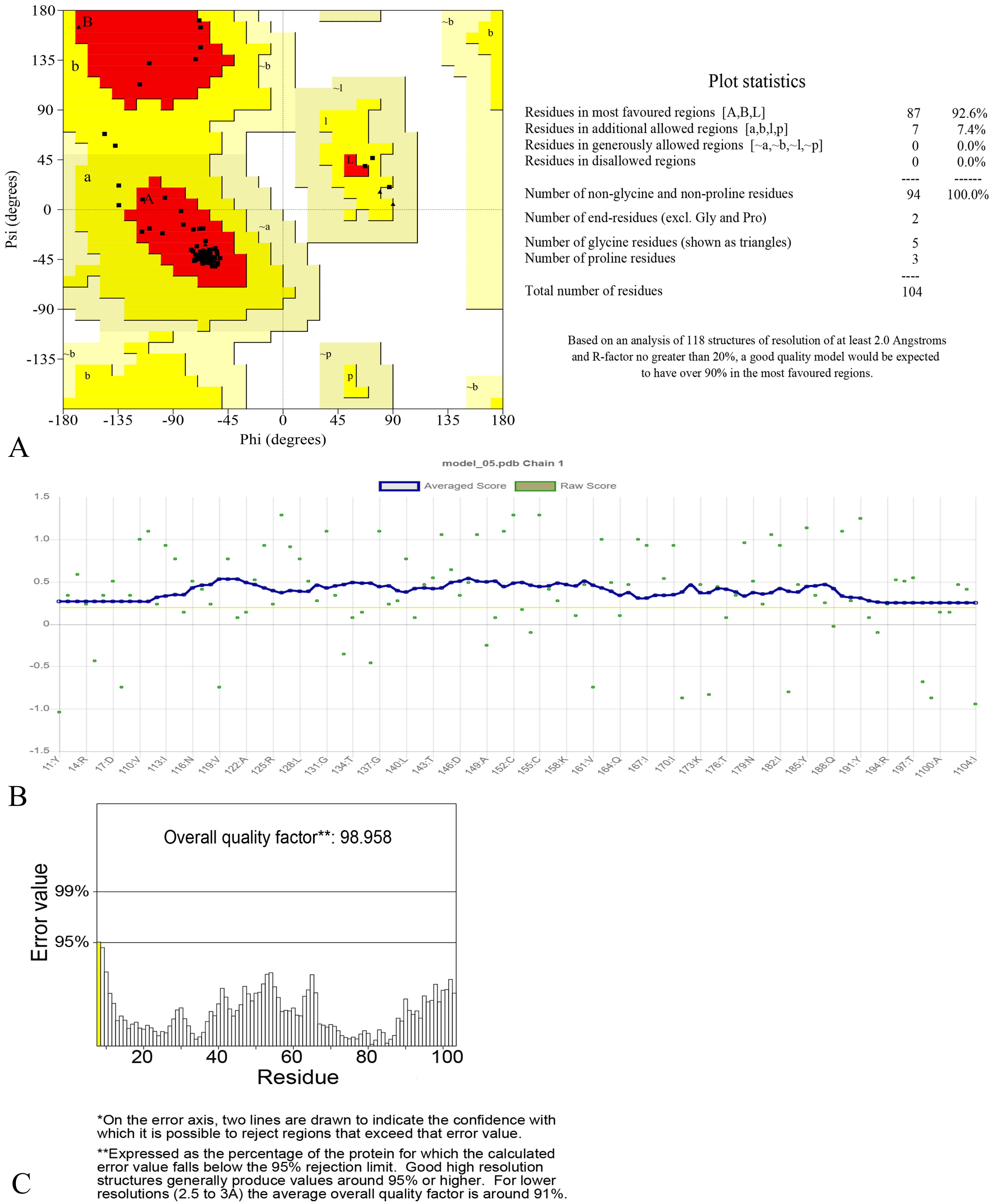

Figure S1. Quality evaluation of the SnitCSP2 model. (A) Ramachandran plot; (B) Verify-3D; (C) ERRAT.
